# Cardiorespiratory Performance in Kidney and Liver Transplant Recipients: The Dilemma to Combine Lifestyle and Fitness

**DOI:** 10.3390/jfmk9010044

**Published:** 2024-02-29

**Authors:** Giovannino Polara, Alessio Montagnoli, Roberto Palazzo, Melissa Orlandi, Gabriele Mascherini, Marco Corsi, Edoardo Falconi, Laura Stefani

**Affiliations:** 1 Sports Medicine Center, Department of Experimental and Clinical Medicine, University of Florence, 50134 Florence, Italy; 2 Department of Experimental and Clinical Medicine, University of Florence, 50134 Florence, Italy

**Keywords:** body composition, kidney transplant, liver transplant

## Abstract

It is widely demonstrated that moderate-intensity exercise is associated with improved fitness in non-communicable chronic diseases. However, there are no specific guidelines available for transplant recipients. Body composition is closely linked to exercise capacity, typically estimated by cardiopulmonary testing, but its potential correlation with cardiovascular health outcomes has not been investigated yet. This study aims to evaluate and compare cardiorespiratory performance and body composition in two groups of liver and kidney transplant recipients. A mixed group of transplant recipients (10 kidney and 15 liver) participating in a lifestyle reconditioning program through unsupervised physical exercise prescription was examined. Both groups were assessed using bioimpedance analysis (BIA), lifestyle, and physical activity levels by IPAQ questionnaire and cardiopulmonary testing (CPET). The two groups differed by IPAQ examination: liver transplant patients practiced more physical activity. Statistically significant differences were found in peak VO_2_/HR (oxygen pulse), which was higher in the kidney group compared to the liver group (15.63 vs. 12.49 with *p* < 0.05). Body composition did not show significant differences in BMI and the percentage of FM/FFM (FFM: 78.04 ± 7.7 in Kidney T vs. 77.78 ± 7.2 in Liver T). Systolic pressure measured at the peak was significantly higher in the liver group (162.6 vs. 134 with *p* < 0.01). The correlation between the CPET and BIA parameters showed a positive VO_2_ max and FFM mass trend. The results suggest differences in cardiorespiratory fitness between the two populations of solid organ transplant recipients despite not being related to the physical activity level. The data support the importance of body composition analysis in sports medicine and the prescription of physical exercise, especially considering the potential correlation with VO_2_ max, even though home-based exercise does not seem to alter it substantially. BMI does not appear to be a determinant of cardiovascular performance. Other determinants should be investigated to understand the differences observed.

## 1. Introduction

Solid organ transplant recipients are a unique population that has recently become involved in exercise prescription programs [1,2,3]. Exercise has been shown to offer numerous benefits, particularly for the muscular and cardiovascular systems. It can also slow the progression of chronic degenerative diseases [4,5,6,7,8,9]. Moreover, considering the evolving landscape of medical advancements and personalized medicine, incorporating innovative approaches into exercise prescription guidelines for transplant recipients is crucial. Tailoring recommendations based on individual medical histories and evolving health conditions could further optimize the effectiveness of exercise interventions.

Specifically challenging lifestyle through reconditioning could have a strong impact on quality of life, allowing this population to regain a normal lifestyle. From a larger point of view, a potential positive impact on the social cost could be hypothesized, considering the longer life expectancy but higher exposure to comorbidities and complications. However, specific guidelines for exercise prescription in this group are currently lacking. Some research has been performed in this context with evidence of a progressive, positive impact of physical exercise in transplant subjects [6,10,11]. Especially in this population, the risk of developing metabolic syndrome remains high after transplantation, and therefore, physical activity can be proposed if individually tailored at a moderate intensity [10,11]. Physical inactivity is a crucial driver of progression and adverse outcomes in liver diseases as well as those of the kidney [8,10]. Long-term exposure to immunosuppressive drugs and reduction in the strength and power of muscles can contribute to a potential decrease in cardiovascular performance, especially in the VO_2_ max parameter measured by cardiopulmonary testing [11,12]. Furthermore, transplant patients have long been exposed, in the period preceding the transplant, to high levels of toxic catabolites due to the patient’s renal or hepatic insufficiency. Body composition evaluation serves as the baseline assessment before initiating exercise prescription programs [13,14] and to non-invasively detect any body composition dysfunction, especially in those whose water distribution is impaired and anomalous. Bioimpedance analysis (BIA) is the best method to investigate water distribution and nutrition reduction disturbances among the anthropometric parameters. The potential relationship between body composition and cardiovascular performance has yet to be investigated. The investigation of this aspect could reasonably clarify reduced performance, and difficulties in constantly adhering to exercise programs is proposed, particularly among frail subjects. This study aimed to analyze and compare body composition and cardiopulmonary exercise test (CPET) data between kidney and liver transplant patients and explore any correlations between the body composition parameters and CPET parameters. This information may prove valuable for tailoring exercise prescriptions for solid organ transplant patients and enhancing their overall health and well-being.

## 2. Materials and Methods

A total of 25 transplant patients (15 liver transplants and 10 kidney transplant subjects, aged 60 years or older) who were regularly monitored for the exercise prescription program at the Sports Medicine clinic of the University Hospital of Florence were included in this study. The investigation took place from February 2020 to October 2020. All participants were at least one-year post-transplant and were clinically stable. All participants provided informed consent, and the same tests were conducted for each subject throughout the study. The inclusion criteria were to have been transplanted for at least one year and clinically stable (e.g., the absence of liver-related complications in the previous six months, including acute rejection episodes and increased serum transaminases two times the upper limit). The exclusion criteria were combined transplantation, re-transplantation, physical limitations, cardiovascular contraindications to exercise, and psychiatric or severe debilitating neurological disorders. All participants had mild or moderate hypertension and were on anti-hypertensive medications (such as calcium channel blockers, ACE inhibitors, or ARBs) as well as immunosuppressive therapy, including calcineurin inhibitors (Ciclosporin or Tacrolimus) in combination with Mycophenolate or Everolimus and steroids (Methylprednisolone). None of the participants had major arrhythmias the few months before the study. The physical exercise program consisted of a mixed physical activity regimen, including aerobics and resistance training at least three times a week for 30 min of aerobic exercise at an intensity of approximately 60% of the maximum heart rate in an unsupervised manner. The heart rate range for aerobic activity was individually determined using the Karvonen formula based on the heart rate range of the two thresholds [15]. The adherence to the physical activity program was measured by the International Physical Activity Questionnaire (IPAQ) [16], interpreting values <700 Mets/k/w as sedentary behavior, values in the range of 700 to 2200 as compatible with moderate activity levels, and values >2200 as very active. The data obtained by the IPAQ were compared to the physical activity level monitored in the specific lifestyle application of the patient’s mobile phone, where the step numbers are registered. After a clinical and anamnestic evaluation, the participants’ body composition was assessed by measuring nutritional and hydration status in a resting condition. A cardiopulmonary exercise test (CPET) that included various cardiovascular and respiratory parameters was also conducted.

### 2.1. Bioimpedance Measurement and Body Composition Assessment

Bioimpedance analysis (BIA) is a safe, fast, non-invasive, and cost-effective technique used to estimate body composition in clinical practice and population studies [17]. The BIA operates on the principle of passing a low-intensity alternating electric current (approximately 800 μA) at 50 kHz through the human body, which travels at different speeds depending on its composition. The body, mainly composed of water and ions, with “lean” tissues containing a higher quantity than “fat” tissues, facilitates this. Lean tissues, such as bone and muscle, have a more hydrated cellular population than adipocytes in adipose tissue, allowing BIA to estimate body composition with reasonable accuracy. The key parameter of this technique is impedance (Z), determined by two elements: resistance (R), representing the ability of biological structures to resist the flow of current, and reactance (Xc), indicating the force opposing current passage due to cellular membranes and cellular mass (BCM). Another related value is the phase angle (PhA), which describes the relationship between resistance (R) and reactance (Xc). This parameter depends on the subject’s hydration status (and the ECW/ICW ratio) and their cellular mass (BCM), offering insights into membrane integrity and cellular functionality. The application of BIA is not only restricted to clinical settings but extends to population studies, showcasing its versatility and relevance in diverse research contexts. An Akern BIA 101 BIVA^®^ PRO bioimpedance analyzer (Pisa, Italy) was used in this study. The standard procedure required the patient to lie in a supine position, at rest for a few seconds, on a flat surface, free of any metal objects, with their arms and legs spread apart at 30° and 45°, respectively. The following values were measured to assess body composition before the BIA analysis: Weight (kg) and height (m) were measured using a mechanical scale with a stadiometer in an upright position, without shoes, with an approximation to the nearest 0.1 (kg or m) for excess or deficit. Body Mass Index (BMI) (kg/m^2^) was calculated as the ratio of weight (kg) to height squared (m^2^). For body composition, the following parameters were considered: lean mass (FFM), fat mass (FM), cell mass (BCM), intracellular water (ICW), extracellular water (ECW), total body water (TBW) expressed as a percentage (%), phase angle (PhA) expressed in degrees (°), and the Hydragram hydration index, expressed as the ratio between TBW and FFM [18].

### 2.2. Cardiopulmonary Test (CPET)

The CPET was conducted according to established guidelines [11,12,19,20] using an electromagnetic brake cycle ergometer (Ergoline) and a specific gas measurement machine (COSMED Quark CPET, Albano Laziale, Rome, Italy). Each participant was instructed to avoid strenuous physical exertion the day before the test and to abstain from consuming solid foods or carbohydrate-rich drinks for three hours before the test. The test was performed in the morning under controlled conditions (temperature: 18–24 °C; humidity: 30–60%). The ramp protocol for cardiopulmonary testing was tailored based on gender and body composition to aim for muscle exhaustion between 8 and 12 min. Participants wore an oro-facial mask connected to a gas-measuring device. Exhaled CO_2_ and O_2_ consumption were measured breath by breath. The lowest possible increase in watts (1, 2, or 5 watts) was set for each ramp to achieve the most linear increase in load and, therefore, a more physiological response. After 3 min of warming up by cycling without load at 50 rpm, the test followed these steps: at the start of the actual effort, cycling was required at a cadence between 60 and 80 rpm until muscle exhaustion. The test concluded when participants could no longer maintain their cycling cadence despite verbal encouragement. The test was considered maximal if at least two of the following criteria were met: Respiratory Exchange Ratio (RER) > 1.10, maximum heart rate > 85% according to age, and a plateau in oxygen consumption (increase < 150 mL·min^−1^) in the last 30 s of the test. The test was stopped early in the presence of cardiovascular signs and symptoms (complex ventricular arrhythmias, drops in systolic blood pressure, dizziness, etc.). Continuous monitoring included a 12-lead ECG and oxygen saturation. During the test, various parameters were measured, including oxygen consumption (VO_2_), carbon dioxide production (VCO_2_), tidal volume (VT), respiratory rate (RF), minute ventilation (VE), heart rate (HR), and workload (WR). The lactate threshold was determined using the V-slope and ventilator equivalents approach. Other variables analyzed included the relationship between oxygen consumption and heart rate (VO_2_/HR, a measure of stroke volume), the relationship between oxygen consumption and workload (VO_2_/W slope, a measure of circulatory efficiency), and the product of VO_2_ peak (mL/kg/min) and systolic blood pressure (a measure of circulatory strength).

### 2.3. Statistical Analysis

Data are expressed as the mean and standard deviation. An analysis was conducted using an unpaired Student’s *t*-test, and the statistically significant value was set at *p* < 0.05. A linear correlation test was also performed between the mentioned data.

## 3. Results

The results obtained were assessed as the averages between the two groups. Subsequently, linear correlations of the body composition parameters with the cardiopulmonary test data of the two groups were conducted. All the subjects were asymptomatic for palpitation or chest pain and in stable clinical conditions. The blood pressure values, in rest conditions, were in the normal range without significant differences between kidney and liver transplant subjects (Table 1). All of them were overweight (BMI > 25), and no significant differences were observed in the two groups (Table 1). The physical activity monitoring by IPAQ demonstrated that the liver transplant subjects were more active than the kidney ones (Table 1). The range of the physical workload for the liver transplantation patients was in the range of moderate activity level (700 to 2200 Mets/h/w), while in the kidney transplant group, it was compatible with sedentary activity (<700 M/h/w). The body composition parameters showed otherwise average values, especially for the FFM percentage (Table 2). The hydration analysis demonstrated a slight increase in the total body water as expressed by the angle phase value being just a little under the normal range associated, otherwise to a higher value of TBW (Table 2). It is known that values lower than 5° may indicate cell damage and/or cell membrane rupture [6,14,20], which is a trend evident in this study in kidney transplant recipients (5.85 ± 1.2) if compared to liver transplant recipients (6.3 ± 0.9). The water distribution was, therefore, normal, with a predominant level in the intracellular site. No significant differences were found for the other parameters (Table 1).

The hydration and nutrition status were compatible with normal values in all patients. The phase angle (PhA) values (Table 2) were slightly lower in the kidney transplant patients than in the liver patients.

Some statistically significant differences were, on the contrary, observed between the two groups, especially concerning the peak systolic blood pressure, measured at the maximal effort, which was higher in liver transplant recipients (Table 3). The peak VO_2_/HR, as an expression of cardiovascular performance, showed a higher value in kidney transplant recipients (Table 3). This aspect could be interpreted in the first line based on the slight difference in body composition, with higher FFM values partially confirmed by the positive correlation trend.

**Figure 1:**
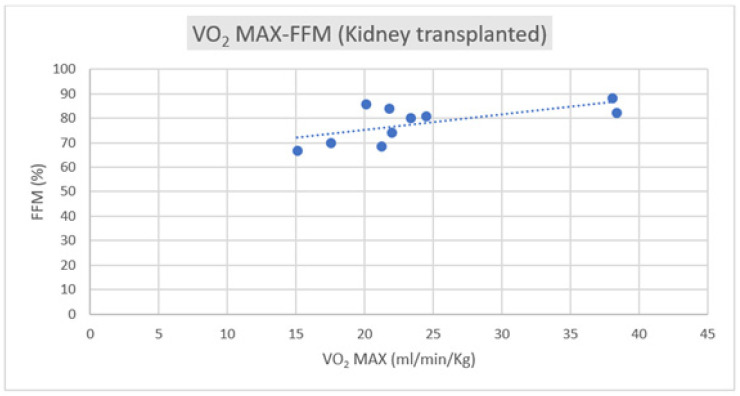
Correlation of VO_2_ max–FFM in kidney transplant subjects (R = +0.5185).

**Figure 2:**
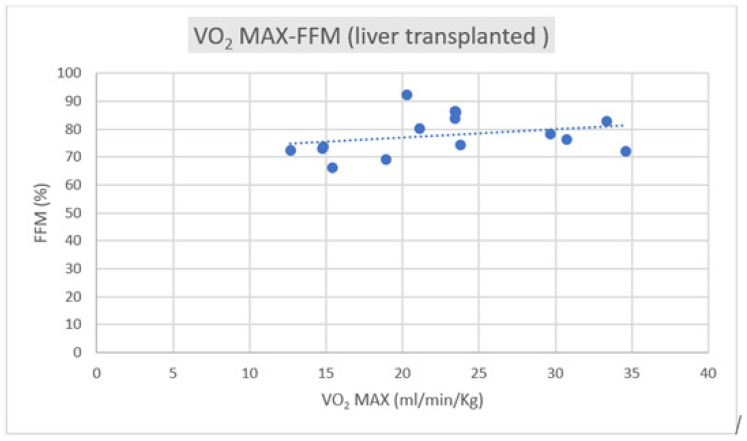
Correlation of VO_2_ max–FFM in liver transplant subjects (R = +0.2089).

Regarding the correlation between CPET parameters and body composition, we observed a positive correlation trend between VO_2_ max and FFM in both groups, as depicted in Figure 1 and Figure 2.

Furthermore, the maximal oxygen uptake (VO_2_ max) demonstrated a parallel negative correlation trend with fat mass (R—0.42692), with a linear correlation coefficient of R = −0.42692. These correlations align with findings from the existing literature, indicating that VO_2_ max is associated with better body composition while being negatively related to the adipose component [6,21,22]. This represents a novel aspect not previously reported in studies, especially within the transplant population.

## 4. Discussion

Lifestyle reconditioning is important to reduce cardiovascular risk factors in many metabolic chronic diseases, including post-transplantation. Changes, especially in cardiorespiratory function and metabolic profile, have been largely observed in the presence of comorbidities, like coronary artery disease, hypertension, and diabetes, and also post-transplantation [1,2,3,4,5,6,7,8,9,10,21,22,23]; however, other specific determinants, such as the body composition parameters, have not been yet investigated. Prolonged sedentarism and potential cardiotoxicity due to the long-term immunosuppression therapy increase the eventual impairment of the system–diastolic function, reducing the cardiorespiratory performance and the possibility of following constant training. To restore adequate global fitness to maintain the transplanted organs’ normal function, attention to cardiorespiratory and body fitness appears fundamental. Adherence to a correct lifestyle, in terms of an adequate Mediterranean diet and number of daily steps, is the basis for reducing cardiovascular risk factors; however, effective fitness is the next step to continuing regular physical activity [22,23,24,25]. Fitness means that conditions of well-being and body performance permit progressive workload training. The body composition analysis is one of the drives to manage the mixed endurance and resistance training plan. The hydration status in transplant subjects is often worsened by the excessive compartmentalization of water, mainly distributed in the extracellular district. This aspect can contribute to an augmented risk of particularly dehydrated muscle injuries. In parallel, a potential sarcopenic condition due to long-term sedentarism with an effective reduction in fatty-free mass can reduce exercise tolerance, compromising regular training, even at moderate intensity. The different behavior of these parameters, especially the relationship with the cardiovascular parameters in diverse transplant patients, is an object of interest. Generally, moderate-intensity physical activity, whether supervised or unsupervised, has been demonstrated to have numerous benefits in reducing cardiovascular risk factors and overall mortality [26,27,28,29]. These benefits have also been observed in populations of patients undergoing organ transplantation [6,30]. Body composition has been demonstrated to have a crucial role in physical performance since the pre-transplantation period. Fat-free mass (FFM) accounts for about 80-85% of total body weight and is closely linked to basal energy expenditure and increases with physical activity. At the same time, it naturally decreases with age and a sedentary lifestyle. In contrast, a higher percentage of fat mass is associated with conditions such as diabetes, cardiovascular disease, certain types of cancer, and physical disability [27,28,29,30]. A phase angle value lower than 4.9° is associated with a condition known as sarcopenia [17,31,32,33], characterized by a quantitative and qualitative deficiency in muscle tissue. This phenomenon has also been observed in the pre-transplant period, for example, in liver cirrhosis [34], kidney transplantation, and in heart failure [35]. In the two populations studied, the BMI and body composition data were substantially similar, except for cellular water compartmentalization, which was slightly less favorable in the kidney transplant group. However, this difference did not reach statistical significance. On the contrary, the lifestyle is very different considering the estimation by the IPAQ questionnaire. The liver transplant subjects are more active than the kidney transplant recipients. These data contrast with the CPET results, such as peak VO_2_/HR and peak systolic blood pressure, which are significantly different in the two groups. The oxygen pulse and VO_2_ max were higher in the kidney group despite a lower FFM level and reduced weekly physical activity. It is reasonable that other determinants could impact this peculiar behavior, such as the time to transplantation and, therefore, the time to exposition to the cardiotoxicity of the immune suppressive therapy or the adherence to a correct diet program. None of these aspects are investigated in the present study, and they will be objects of interest in the future. The results revealed a linear positive correlation between maximum oxygen consumption (VO_2_ max) and fat-free mass and a negative correlation of the same parameter with fat mass. These trends were more pronounced in the kidney group, where the VO_2_ max was higher. This implies that a greater lean component, and consequently, a larger quantity of muscle, leads to greater strength in the lower limbs. The peak oxygen pulse did not appear to correlate significantly with body composition in the overall sample. However, it did show a positive correlation with lean body mass in the kidney group. This aspect could be interpreted in terms of a low level of training, considering they are under unsupervised exercise. The correlation between the gold standard of cardio-pulmonary performance (VO_2_ max) and body composition underscores the latter’s significance in facilitating physical activity and prescribing physical exercise as a medical–sports therapy to reduce cardiovascular risk and overall mortality.

### 4.1. Study Limitations

This study has several limitations that should be acknowledged. These include a relatively small sample size and confounding factors that must be considered. 

In particular, the low number of subjects investigated could represent the major limit to having significant evidence of correlations between the body composition and myocardial performance. It is also important to underline that a long-term follow-up could contribute to providing more evidence of a regular and constant adherence to the training proposed, actually evaluated exclusively by a simple mobile phone application and not by dedicated accelerometry. Other confounding factors encompass variations in pharmacological therapies administered to the two groups of patients, potential cardiotoxic and myotoxic effects of specific medications, variances in the underlying pathology that necessitated the transplant, the duration since the transplant at the time of evaluation, and the participant’s level of physical activity. A more comprehensive understanding of their contributions to the observed differences in the cardiopulmonary tests between the two groups under consideration will require multivariate analysis. A more comprehensive approach is therefore needed to clarify the role played by body composition parameters in these differences.

### 4.2. Future Directions

Numerous studies have clarified the positive role of exercise prescription in transplant subjects. However, confounding factors like the distance from the transplantation date as an expression of potential progressive damage due to a longer exposure to drug treatment for immunosuppressive therapy are not yet evaluated. In parallel, the physical activity level measured by the IPAQ could also play a role in defining the impact of sedentarism in enhancing cardiovascular performance. Some other aspects regarding energy availability, such as net differences between the effective intake and the output due to the daily energy expenditure, have yet to be investigated. This evaluation could represent the essential investigation of transplant subjects in whom a reduced level of performance could be due to reduced energy. Other studies will be necessary to understand the determinants of physical activity performance.

## 5. Conclusions

Transplant subjects are a particularly frail category, with a high cardiovascular risk profile potentially dependent on reduced muscle performance as a consequence of prolonged sedentarism and drug toxicity. Consequently, the long-term follow-up of these patients places a significant emphasis on assessing body composition, especially when exercise programs, often tailored, are required to restore muscle power and strength and maintain fitness conditions. The body composition represents, therefore, one of these patients’ principal objectives of interest. Despite the slight evidence emerging from the results obtained, the relationship between bioelectrical parameters and physical performance in transplant recipients is a new direction of study never investigated before, especially in this population. The present study does not investigate the role of PSA values that reflect greater cellularity support in physical performance close to maximum individual effort. In some cases, the data are suggestive and in agreement with other studies to support the relevant role of lifestyle correction as a therapeutic intervention that is important to promote in post-transplant subjects [3]. Lifestyle intervention is usually proposed as diet correction and/or a combination of physical activity. Despite the differences between the two groups investigated, particularly in the weekly level of physical activity, this aspect does not seem sufficient to induce greater performance. Other aspects related to energy availability and the specific training need to be studied in the future as well other confounding factors, such as differences in immunosuppressive regimens, time since transplantation, and underlying disease etiology, that are not considered in the present study. These missing data limit the generalization of the preliminary results obtained. Cardiopulmonary exercise testing (CPET) is commonly employed in medical sports evaluations and for prescribing physical exercise, as it provides essential insights into cardiorespiratory performance and potential exercise limitations. Body composition analysis is a sensitive and widely used clinical method for monitoring training progress in the general population and organ transplant recipients. A notable suggestion from this study is the potential implementation of resistance exercises in exercise prescription programs for transplant subjects. Recognizing the importance of integrating CPET assessments with body composition parameters, this study underscores the need for a holistic approach to comprehensively understand and precisely measure cardiorespiratory performance in solid organ transplant recipients. This multifaceted perspective is imperative for tailoring effective exercise programs and optimizing transplant recipients’ overall health and well-being. In essence, this study paves the way for future research endeavors to refine and expand our understanding of the intricate interplay between bioelectrical parameters, lifestyle interventions, and exercise outcomes in this unique population.

## Figures and Tables

**Table 1:** General data of the two groups of transplant subjects.

N Subjects (25)	Kidney (10)	Liver (15)	*p* Value
Age	57.7 ± 5.0	61.3 ± 10	NS
Weight (Kg)	80.2 ± 14	78.2 ± 11.0	NS
Height (cm)	178.0 ± 7	171.0 ± 6	NS
BMI (kg/m^2^)	25.8 ± 3.3	26.5 ± 3.3	NS
DBP (mmHg)	81.6 ± 11.0	81.3 ± 8.5	NS
SBP (mmHg)	125.0 ± 13.0	124.0 ± 16.0	NS
IPAQ Mets/h/W	450.3 ± 30.5	1430.0 ± 81.0	<0.01

Legend: BMI: Body Mass Index; DBP: Diastolic Blood Pressure; SBP: systolic blood pressure; IPAQ: International Physical Activity Questionnaire.

**Table 2:** Hydration and nutrition analysis of liver and kidney transplant recipients.

Body Composition	Kidney	Liver	*p*-Value
PhA (°)	5.8 ± 1.2	6.3 ± 0.9	NS
TBW (%)	57.63 ± 6.2	57.1 ± 5.3	NS
ECW (%)	47.0 ±5.9	44.3 ± 4.0	NS
ICW (%)	52.9 ± 5.9	55.6 ± 4.0	NS
FFM (%)	78.0 ± 7.7	77.7 ± 7.2	NS
FM (%)	21.9 ± 7.7	22.2 ± 7.2	NS
BCM (%)	52.3 ± 6.4	55.2 ± 4.0	NS
BCMI (%)	10.5 ± 1.8	11.3 ± 1.1	NS

Legend: PhA: phase angle; TBW: total body water; ECW: extracellular water; ICW: intracellular water; FFM: fat-free mass; FM: fat mass; BCM: Body Cell Mass; BCMI: Body Cell Mass Index.

**Table 3:** Comparison of CPT parameters between renal and liver transplant recipients.

CPET	Kidney (10)	Liver (15)	*p* Value
VO_2_ max (mL/min/Kg)	24.2 ± 7.8	22.6 ± 6.9	NS
VO_2_ max (%)	83.5 ± 23.6	83.4 ± 22.2	NS
VO_2_ 1 Threshold (mL/min/Kg)	12.5 ± 6.2	12.4 ± 3.5	NS
VO_2_ 2 Threshold (mL/min/Kg)	18.7 ± 6.2	17.0 ± 4.7	NS
HR peak (bpm)	126.7 ± 19.7	143.7 ± 29.1	NS
VO_2_/HR peak (mL/bpm)	15.6 ± 4.0	12.4 ± 2.9	<0.03
METs/peak	7.2 ± 2.2	6.6 ± 1.9	NS
Watt/peak	145.2 ± 66.2	127.2 ± 45.2	NS
VE/VCO_2_ slope	34.9 ± 7.0	34 ± 5.0	NS
SBP Peak (mmHg)	134.0 ± 17.1	162.6 ± 19.3	<0.001
DBP Peak (mmHg)	81.5 ± 10.0	78.6 ± 12.1	NS
HR 1 threshold (bpm)	89.8 ± 16.7	97.4 ± 14.5	NS
HR 2 threshold (bpm)	108.5 ± 16.9	120.2 ± 21.1	NS

Legend: SBP: systolic blood pressure; DBP: Diastolic Blood Pressure; HR: heart rate; VO_2_: Ventilatory Oxygen; VE/VCO_2_ (minute ventilation/carbon dioxide production) Ventilatory Exchange.

## Data Availability

Data can be obtained from Laura Stefani upon reasonable request at laura.stefani@unifi.it.
